# Association between multiple coagulation-related factors and lymph node metastasis in patients with gastric cancer: A retrospective cohort study

**DOI:** 10.3389/fonc.2023.1099857

**Published:** 2023-02-23

**Authors:** Wenhao Qiao, Shengxu Sha, Jiyuan Song, Yuezhi Chen, Guodong Lian, Junke Wang, Xinxiu Zhou, Lipan Peng, Leping Li, Feng Tian, Changqing Jing

**Affiliations:** ^1^ Department of Gastrointestinal Surgery, Shandong Provincial Hospital, Shandong University, Jinan, Shandong, China; ^2^ Department of Gastrointestinal Surgery, Shandong Provincial Hospital Affiliated to Shandong First Medical University, Jinan, Shandong, China

**Keywords:** gastric cancer, lymph node metastasis, maximum amplitude, prognostic factor, tumor N stage, tumor T stage

## Abstract

**Background:**

Patients with tumors generally present with accompanying activation of the coagulation system, which may be related to tumor stage. To our knowledge, few studies have examined the activation of the coagulation system in reference to lymph node metastasis within gastric cancer. This study aimed to investigate the correlation between multiple coagulation-related factors and lymph node metastasis in patients with gastric cancer after excluding the influence of tumor T stage.

**Materials and methods:**

We retrospectively evaluated the relationship between lymph node metastasis and coagulation-related factors in 516 patients with T4a stage gastric cancer. We further analyzed influencing factors for lymph node metastasis and verified the predictive value of maximum amplitude (MA, a parameter of thromboelastography which is widely used to assess the strength of platelet-fibrinogen interaction in forming clots) in reference to lymph node metastasis.

**Results:**

Platelet counts (P=0.011), fibrinogen levels (P=0.002) and MA values (P=0.006) were statistically significantly higher in patients with T4a stage gastric cancer presenting with lymph node metastasis than in those without lymph node metastasis. Moreover, tumor N stage was statistically significantly and positively correlated with platelet count (P<0.001), fibrinogen level (P=0.003), MA value (P<0.001), and D-dimer level (P=0.010). The MA value was an independent factor for lymph node metastasis (β=0.098, 95% CI: 1.020-1.193, P=0.014) and tumor N stage (β=0.059, 95% CI: 0.015-0.104, P=0.009), and could be used to predict the presence of lymph node metastasis in patients with gastric cancer (sensitivity 0.477, specificity 0.783, P=0.006). The independent influencing factors for MA value mainly included platelet levels, fibrinogen levels, D-dimer and hemoglobin levels; we found no statistically significant correlations with tumor diameter, tumor area, and other evaluated factors.

**Conclusion:**

We conclude that MA value is an independent influencing factor for lymph node metastasis and tumor N stage in patients with T4a stage gastric cancer. The MA value has important value in predicting the presence or absence of lymph node metastasis in patients with gastric cancer.

**Clinical trial registration:**

http://www.chictr.org.cn, identifier ChiCTR2200064936.

## 1 Introduction

Gastric cancer is a major global health problem. Worldwide, gastric cancer is the fifth most prevalent cancer, and presents with the fourth highest mortality rate (1). Lymph node metastasis is one of the most important influencing factors for poor prognosis in gastric cancer (2). A large number of studies have shown that, in addition to factors such as tumor length, tumor short diameter, tumor area, and histopathologic grade, the ratio of preoperative platelets/lymphocytes and neutrophils/lymphocytes as well as various inflammatory indicators may also be related to lymph node metastasis in patients with gastric cancer (3, 4). Moreover, some previous studies reported that coagulation factors may mediate invasion and migration in gastric cancer (5), and additional studies have found that some coagulation factors (such as platelet and fibrinogen levels) may be related to tumor stage in gastric cancer (6, 7). Hence, evidence to date demonstrates an inseparable connection between gastric cancer and blood coagulation.

Coagulation is a highly conserved process that involves the formation of blood clots (thrombi) at the site of the injury, leading to hemostasis. The coagulation process mainly comprises platelet activation, adhesion, and aggregation, as well as deposition and maturation of the fibrin network (8–10). Coagulation-related events in the tumor stroma and local microenvironment (e.g., platelet activation, adhesion, and aggregation, as well as deposition and maturation of the fibrin network and fibrinolysis) may be activated in the presence of tumors, and the degree of activation has been associated with tumor cell growth and metastasis (11–14). Activation of the coagulation system, including platelet activation, can also induce the production of tumor growth factors and can lead to tumor metastasis (15). These findings all reflect the inseparable relationship between tumor development/progression and coagulation indicators. However, there are few studies addressing whether coagulation-related factors are associated with lymph node metastasis in gastric cancer, and most existing studies do not exclude the influence of tumor T stage within their study design.

In this study, we enrolled 516 patients with T4a stage gastric cancer in order to explore the correlation between lymph node metastasis and coagulation-related factors through comparisons oncological and coagulation-related factors. We additionally analyzed the predictive value of maximum amplitude (MA, a parameter of thromboelastography which is widely used to assess the strength of platelet-fibrinogen interaction in forming clots) in gastric cancer patients with or without lymph node metastasis.

## 2 Materials and methods

### 2.1 Patients

We initially selected a total of 1128 patients with primary gastric cancer who presented at Shandong Provincial Hospital between January 2018 and January 2022, and retrospectively collected their clinical and pathological data.

The inclusion and exclusion criteria for the present study are as follows. Namely, we enrolled (1) patients with primary gastric malignant tumors, (2) patients not receiving preoperative neoadjuvant chemotherapy, (3) patients in whom no distant metastases, such as liver or lung metastases, were found on preoperative ultrasound and computed tomography (CT) examinations, (4) patients who underwent radical resection of gastric cancer, (5) patients with a postoperative pathology of adenocarcinoma or signet ring cell carcinoma according to the WTO pathological classification, (6) patients who did not take anticoagulant drugs (such as aspirin) prior to surgery, and (7) patients who denied any previous coagulation disorder.

**Figure 1:**
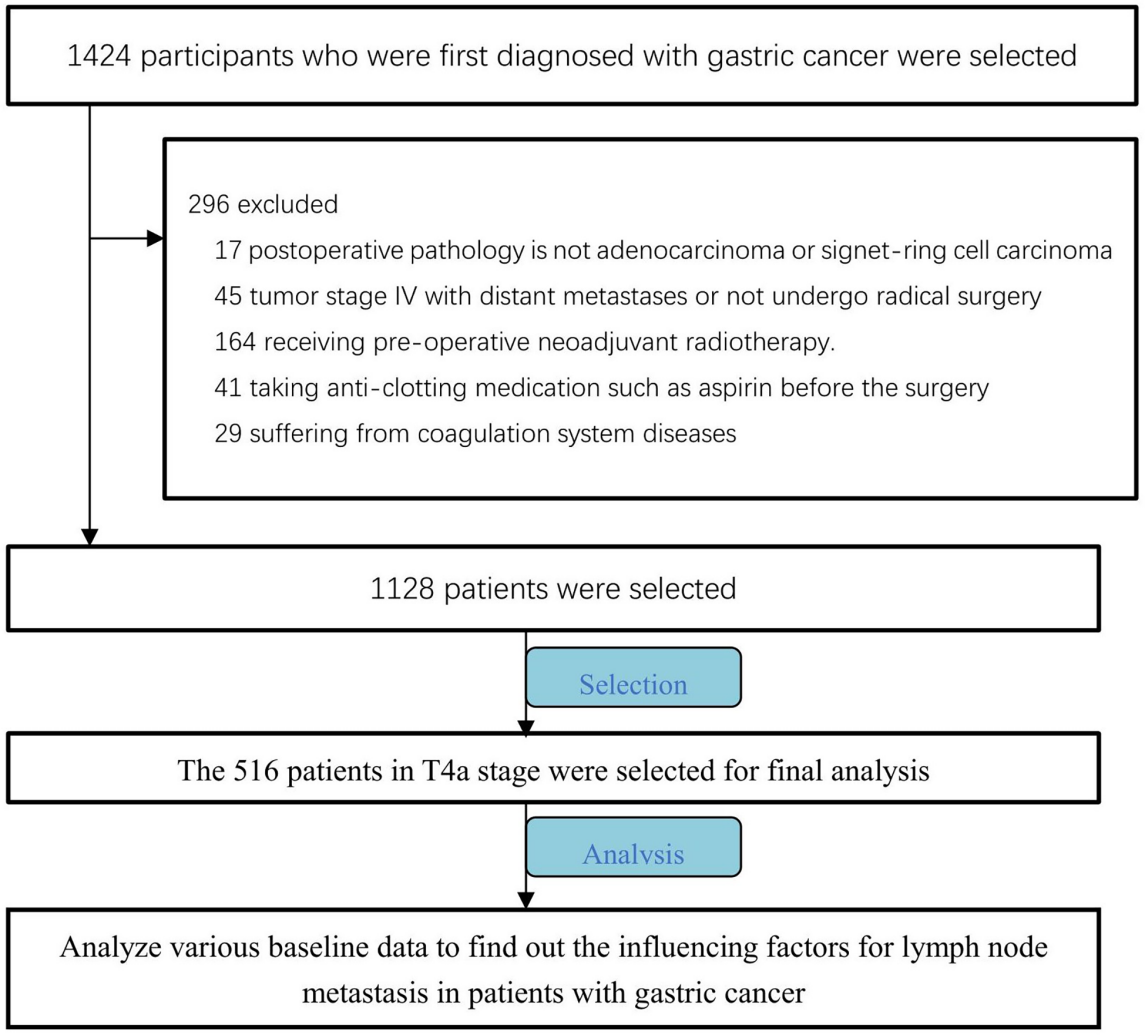
Patient selection flow chart.

Among the identified patients, we selected 516 patients with T4a gastric cancer in order to exclude the influence of tumor T stage, and retrospectively analyzed preoperative baseline characteristics, preoperative laboratory tests, and postoperative pathological results for these patients (**Figure 1**).

This work is reported in accordance with Strengthening the Reporting of Cohort Studies in Surgery (STROCSS) guidelines.

### 2.2 Data collection

We collected data on preoperative baseline indicators, such as age, gender, preoperative Eastern Cooperative Oncology Group (ECOG) score, anesthesia American Society of Anesthesiologists (ASA) score, postoperative pathological results (**Table 1**), and preoperative laboratory test findings (**Table 2**), for the 516 patients with T4a gastric cancer enrolled in this study.

**Table 1:** Clinical participant baseline characteristics.

All patients (N=516)	
Age, years	
Mean (SD)	60.4(10.6)
Median (min, max)	62(28,91)
Gender, n (%)	
Male	372(72.1)
Female	144(27.9)
ECOG PS, n (%)	
0	386(74.8)
1 2	124(24) 6(1.2)
ASA status, n (%)	
I II III	251(48.6) 228(44.2) 37(7.2)
Tumor length, centimeter, cm	
Mean (SD) Median (min, max)	5.8(2.8) 5(0.8,20)
Tumor short diameter, cm	
Mean (SD) Median (min, max)	1.4(0.9) 1.2(0.2,13)
Tumor area, cm^2^	
Mean (SD) Median (min, max)	8.9(12.1) 6.5(0.2,208)
Primary tumor site, n (%)	
Upper Middle Lower Diffuse	101(19.6) 112(21.7) 275(53.3) 28(5.4)
Histopathologic grade, n (%)	
Well differentiated Moderately differentiated Poorly differentiated	7(1.4) 89(17.3) 420(81.4)
WHO classification, n (%)	
Papillary Tubular Mucinous Signet-ring cell Adenocarcinoma, other	2(0.4) 419(81.2) 28(5.4) 38(7.4) 29(5.6)
Vessel invasion, n (%)	
Yes No	206(39.9) 310(60.1)
Neural invasion, n (%)	
Yes No	280(54.3) 236(45.7)
pN, n (%)	
pN0 pN1 pN2 pN3 pN3a pN3b	83(16.1) 85(16.5) 128(24.8) 220(42.6) 210(40.7) 10(1.9)
AJCC/UICC staging, n (%)	
IIB IIIA IIIB IIIC	83(16.1) 213(41.3) 210(40.7) 10(1.9)

AJCC/UICC, American Joint Cancer Committee/Union Internationale Contre le Cancer. The eighth edition (2016) of the AJCC staging system was used.

**Table 2:** Basic statistic data of laboratory examinations for the clinical participants.

Group parameter		N0-N3	N0	N1	N2	N3a	N3b	N+
Patients counts		n=516	n=83	n=85	n=128	n=210	n=10	n=433
White blood cell (x10^9/L) (n=511)	Mean	5.89	5.82	5.97	5.75	5.98	5.71	5.90
	Std	1.71	1.56	1.69	1.70	1.80	1.24	1.74
	Max	14.67	11.96	10.23	14.67	13.25	3.81	14.67
	Min	1.96	3.38	2.75	3.26	1.96	7.79	1.96
Red blood cell (x10^12/L) (n=511)	Mean	4.19	4.22	4.27	4.18	4.17	4.01	4.19
	Std	0.67	0.67	0.72	0.64	0.67	0.72	0.67
	Max	5.92	5.35	5.92	5.55	5.68	5.25	5.92
	Min	2.00	2.43	2.36	2.47	2.00	3.22	2.00
Hemoglobin (g/L) (n=511)	Mean	120.60	124.56	125.05	117.23	119.88	110.30	119.88
	Std	25.45	23.36	25.22	25.76	25.96	21.81	25.77
	Max	171	160	171	164	163	145	171
	Min	44.3	54	62	53	44.3	86	44.3
Platelet count (x10^9/L) (n=511)	Mean	260.40	239.95	241.13	264.36	267.02	396.40	264.13
	Std	77.03	55.87	61.60	83.21	77.51	91.04	79.79
	Max	623	369	529	562	623	559	623
	Min	103	116	103	109	130	238	103
MA value (mm) (n=285)	Mean	60.58	58.03	58.74	60.18	61.87	69.30	61.07
	Std	6.73	4.93	5.85	7.32	6.46	6.41	6.92
	Max	78.8	69.4	71.4	77.4	78.8	75.8	78.8
	Min	45.9	50.9	48.7	45.9	47.0	58.3	45.9
fibrinogen count (g/L) (n=502)	Mean	3.21	2.99	3.22	3.23	3.25	3.82	3.25
	Std	0.69	0.64	0.69	0.63	0.71	0.79	0.69
	Max	6.85	5.1	5.94	5.26	6.85	4.95	6.85
	Min	1.52	1.52	1.93	1.93	1.72	2.80	1.72
D-dimer (mg/L) (n=502)	Mean	0.75	0.58	0.59	0.72	0.88	0.92	0.78
	Std	1.05	0.64	0.74	1.06	1.22	1.36	1.10
	Max	9.80	3.50	5.2	7.1	9.8	4.5	9.8
	Min	0.00	0.00	0.00	0.00	0.10	0.15	0.00
PT (s) (n=502)	Mean	11.91	11.90	11.89	11.79	12.00	11.97	11.91
	Std	1.22	1.14	1.91	0.94	1.06	0.91	1.23
	Max	26.60	17.2	26.6	14.9	15.6	13.8	26.6
	Min	9.50	9.5	10.2	10.1	9.6	11.1	9.6
APTT (s) (n=502)	Mean	30.64	30.20	30.10	30.83	30.87	31.53	30.73
	Std	3.88	4.25	2.88	4.03	3.98	3.77	3.81
	Max	51.4	45.5	36.7	43.6	51.4	40.9	51.4
	Min	21.0	23.0	23.1	21.0	23.6	28.4	21.0
TT (s) (n=502)	Mean	14.52	14.73	14.31	14.57	14.53	13.77	14.49
	Std	1.44	1.58	1.34	1.46	1.41	1.26	1.41
	Max	19.2	18.8	17.7	18.5	19.2	16.4	19.2
	Min	11.7	12	11.7	11.9	11.8	12.2	11.7

This work was approved by the Medical Ethics Committee of the Shandong Provincial Hospital (affiliated with Shandong University, Jinan, China). Informed consent was obtained from all patients. This study was conducted in accordance with the principles of the Declaration of Helsinki.

### 2.3 Statistical analysis

Data are reported as means ± standard deviations. We employed univariate and multivariate analysis to determine the independent influencing factors for lymph node metastasis and tumor N stage in patients with gastric cancer. We constructed a receiver operating characteristic (ROC) curve to evaluate the predictive value of MA in patients with gastric cancer presenting with or without lymph node metastasis, and derived the area under the curve (AUC) and its associated 95% confidence interval (CI). Stepwise linear regression analysis was used to identify independent influencing factors for MA. Statistical analyses were performed using SPSS statistical software (version 22.0; SPSS Inc., Chicago, IL, USA). A two-sided P-value of <0.05 was considered the threshold for statistical significance.

## 3 Results

### 3.1 Baseline patient characteristics

Baseline patient characteristics are presented in **Tables 1** and **2**. The overall male to female ratio was 2.58:1, the mean tumor length was 5.8 ± 2.8 cm, and the mean tumor short diameter was 1.4 ± 0.9 cm. Patients with vascular invasion accounted for 39.9% of the study population, and those with nerve invasion accounted for 54.3% of the population (**Table 1**) The mean platelet count was 260.40 ± 77.03 x10^9^/L, the mean fibrinogen level was 3.21 ± 0.69 g/L, the mean MA value was 60.58 ± 6.73 mm, and the mean D-dimer level was 0.74 ± 1.03 mg/L (**Table 2**).

**Figure 2:**
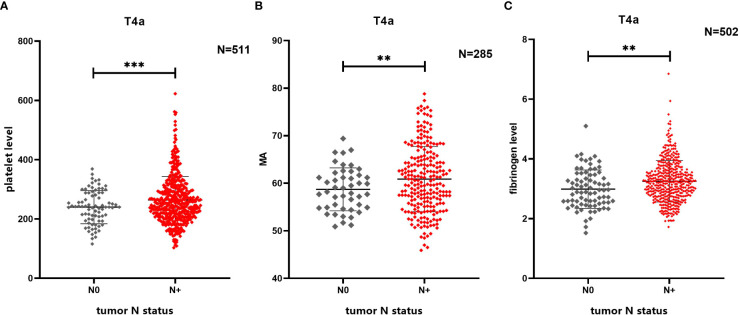
Comparison of differences in multiple coagulation-related factors according to presence or absence of lymph node metastasis. **(A)** Lymph node metastasis in patients with gastric cancer is related to platelet level. **(B)** Lymph node metastasis in patients with gastric cancer is related to MA. **(C)** Lymph node metastasis in patients with gastric cancer is related to fibrinogen level. ** 0.001≤P<0.01, *** P<0.001

Among the patients with gastric cancer initially included in the present study, 292 patients presented with T1 stage cancer (N0: 274, N1: 27, N2: 16, N3a: 2, N3b:0), 151 patients presented with T2 stage cancer (N0: 84, N1: 19, N2: 31, N3a: 17, N3b:0), 169 patients presented with T3 stage cancer (N0: 55, N1: 42, N2: 32, N3a: 39, N3b:1), and 516 patients presented with T4a stage cancer (N0: 83, N1: 85, N2: 128, N3a:210, N3b:10) (**Table 3**). The latter set of patients formed the primary analytic population.

**Table 3:** The number of patients in each T/N stage.

T stage N stage	T1	T2	T3	T4	SUM
**N0**	247	84	55	83	469
**N1**	27	19	42	85	173
**N2**	16	31	32	128	207
**N3a**	2	17	39	210	268
**N3b**	0	0	1	10	11
**SUM**	292	151	169	516	1128

### 3.2 Influencing factors for lymph node metastasis in stage T4a gastric cancer

On univariate analysis, we found that the N+ group (comprising those with lymph node metastasis, including patients with N1, N2, N3a and N3b stage cancer) showed statistically significant differences in tumor length, tumor short diameter, tumor area, primary tumor site, histopathologic grade, vascular invasion, platelet counts, MA values, and fibrinogen levels as compared with the N0 group (those without lymph node metastasis) (**Table 4**). Among these factors, platelet counts, MA values, and fibrinogen levels were determined to be coagulation-related factors.

**Table 4:** Influencing factors of lymph node metastasis(N0/N+) in patients with gastric cancer.

Variables	Univariate			Multivariate		
	EXP(β)	95%CI	p value	EXP(β)	95%CI	p value
Age	0.993	0.971~1.016	0.539			
Sex	1.088	0.640~1.849	0.756			
ECOG	0.873	0.535~1.424	0.585			
ASA	0.984	0.675~1.436	0.935			
Tumor length	1.281	1.145~1.433	0.000	1.314	1.046~1.651	0.019
Tumor short diameter	1.731	1.110~2.700	0.016	1.070	0.332~3.441	0.910
Tumor area	1.089	1.034~1.147	0.001	0.985	0.882~1.100	0.790
Primary tumor site	1.531	1.175~1.996	0.002	1.346	0.873~2.075	0.179
Histopathologic grade	2.907	1.836~4.604	0.000	2.284	1.134~4.602	0.021
WHO classification	0.851	0.661~1.097	0.213			
Vessel invasion	4.330	2.326~8.062	0.000	4.066	1.539~10.742	0.005
Neural invasion	1.500	0.936~2.405	0.092			
WBC	1.029	0.892~1.188	0.695			
RBC	1.930	0.648~1.335	0.696			
HGB	0.992	0.983~1.002	0.128			
Platelet	1.005	1.001~1.008	0.011	0.996	0.990~1.002	0.147
MA	1.007	1.022~1.135	0.006	1.103	1.020~1.193	0.014
D-dimer	1.284	0.910~1.811	0.154			
PT	1.010	0.826~1.237	0.920			
APTT	1.038	0.971~1.109	0.271			
TT	0.892	0.758~1.051	0.171			
Fibrinogen	1.873	1.257~2.790	0.002	1.456	0.705~3.010	0.310

The platelet level in the N+ group was higher than that in the N0 group (264.13 ± 79.79 x10^9^/L *vs*. 239.95 ± 55.87 x10^9^/L, P=0.011), and the MA value in the N+ group was higher than that of the N0 group (61.07 ± 6.92 mm *vs*. 58.03 ± 4.93 mm, P=0.006). The fibrinogen level in the N+ group was also higher than that in the N0 group (3.25 ± 0.69 g/L *vs*. 2.99 ± 0.64 g/L, P=0.002) (**Figure 2**). No statistically significant differences were found in reference to D-dimer levels, prothrombin time (PT), activated partial thromboplastin time (APTT), and thrombin time (TT) (i.e., the additional coagulation-related factors evaluated in this study; see **Supplementary Figure 1**).

Binary logistic regression analysis showed that tumor length, histopathologic grade, vascular invasion, and MA value were independent influencing factors for lymph node metastasis (**Table 4**). Findings for MA value (the only one coagulation-related factor in these factors) were as follows: β=1.103, 95% confidence interval (CI) 1.020-1.193, P=0.014.

### 3.3 Influencing factors for tumor N stage in stage T4a gastric cancer

Univariate analysis showed that ECOG score, tumor length, tumor short diameter, tumor area, histopathologic grade, vascular invasion, neural invasion, platelet counts, MA values, D-dimer levels, and fibrinogen levels statistically significantly differed across tumor N stage (**Table 5**). Among these factors, platelet levels, MA values, D-dimer levels, and fibrinogen levels were determined to be coagulation-related factors.

**Table 5:** Influencing factors of tumor N stage (N0/N1/N2/N3a/N3b) in patients with gastric cancer.

Variables	Univariate			Multivariate		
	EXP(β)	95%CI	p value	EXP(β)	95%CI	p value
Age	-0.010	-0.025~0.005	0.176			
Sex	-0.089	-0.440~0.261	0.617			
ECOG	-0.389	-0.724~-0.054	0.023	-0.127	-0.659~0.406	0.641
ASA	-0.159	-0.411~0.094	0.218			
Tumor length	0.248	0.184~0.312	0.000	0.224	0.114~0.334	0.000
Tumor short diameter	0.296	0.110~0.481	0.002	0.500	-0.154~1.153	0.134
Tumor area	0.033	0.014~0.051	0.000	-0.035	-0.086~0.017	0.190
Primary tumor site	0.126	-0.055~0.308	0.171			
Histopathologic grade	1.219	0.842~1.596	0.000	1.062	0.520~1.604	0.000
WHO classification	-0.012	-0.197~0.172	0.896			
Vessel invasion	1.155	0.817~-1.493	0.000	0.716	0.230~1.203	0.004
Neural invasion	0.511	0.194~-0.828	0.002	0.102	-0.376~0.580	0.675
WBC	0.026	-0.066~0.119	0.578			
RBC	-0.142	-0.379~0.094	0.238			
HGB	-0.006	-0.012~0.000	0.061			
Platelet	0.005	0.003~0.007	0.000	0.002	-0.002~0.005	0.373
MA	0.081	0.047~0.114	0.000	0.059	0.015~0.104	0.009
D-dimer	0.211	0.053~0.389	0.010	0.067	-0.149~0.282	0.544
PT	0.066	-0.069~0.210	0.337			
APTT	0.038	-0.004~0.079	0.077			
TT	-0.036	-0.146~0.175	0.526			
Fibrinogen	0.360	0.123~0.597	0.003	-0.062	-0.452~0.329	0.757

Platelet levels increased statistically significantly with increased tumor N stage (P<0.001). Moreover, platelet levels in patients with N3b stage cancer were statistically significantly different as compared to platelet levels in patients with N0 stage ((P<0.001), N1 stage cancer ((P<0.001), N2 stage cancer ((P<0.001) or N3a stage cancer (P<0.001), and levels in patients with N3a stage cancer statistically significantly differed from those in patients with N0 stage (P=0.006) or N1 stage cancer (P=0.007) as well. Besides, platelet levels in patients with N2 stage cancer statistically significantly differed from those in patients with N0 stage (P=0.021) or N1 stage cancer (P=0.025).

**Figure 3:**
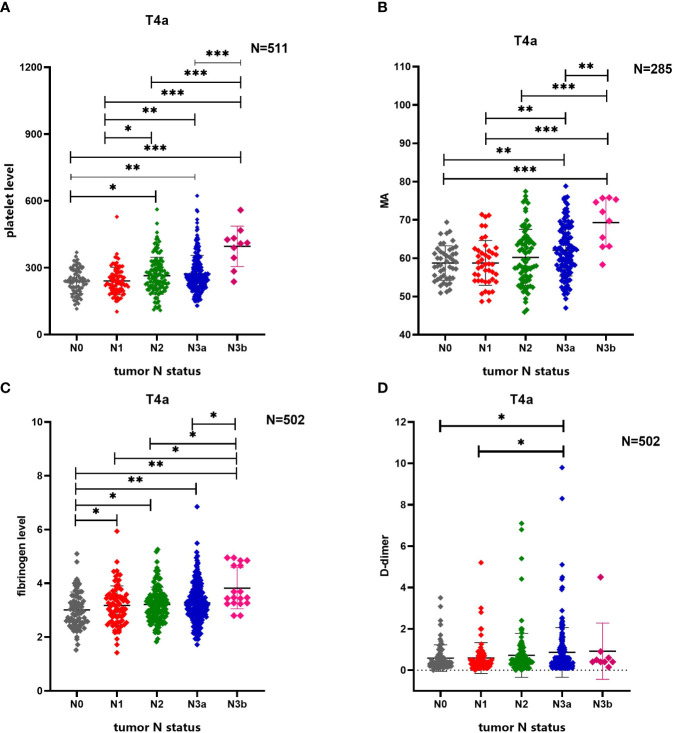
Comparison of differences in multiple coagulation-related factors according to tumor N stage of patients with gastric cancer. **(A)** Tumor N stage of patients with gastric cancer is related to platelet level. **(B)** Tumor N stage of patients with gastric cancer is related to MA. **(C)** Tumor N stage of patients with gastric cancer is related to fibrinogen level. **(D)** Tumor N stage of patients with gastric cancer is related to D-dimer. * 0.01≤P<0.05, ** 0.001≤P<0.01, *** P<0.001.

MA values increased statistically significantly with increased tumor N stage (P<0.001). Moreover, MA values were higher in patients with N3b stage cancer than in those with N0 stage (P<0.001), N1 stage (P<0.001), N2 stage cancer (P<0.001), or N3a stage cancer (P<0.001), and MA values in patients with N3a stage cancer statistically significantly differed from those in patients with N0 stage (P=0.001) or N1 stage cancer (P=0.007) as well. D-dimer levels also increased statistically significantly with an increase in N stage (P=0.017). Moreover, D-dimer levels were higher in N3a stage cancer than in N0 stage (P=0.031) or N1 stage cancer (P=0.031). Fibrinogen levels also increased statistically significantly with an increase in N stage (P=0.007); levels were lower in patients with N0 stage cancer than in those with N1 stage (P=0.034), N2 stage (P=0.015), N3a stage (P=0.003), or N3b stage cancer (P=0.001). Besides, fibrinogen levels in patients with N3b stage cancer statistically significantly differed from those in patients with N1 stage (P=0.012), N2 stage (P=0.012) or N3a stage cancer (P=0.015). (**Figure 3**)

Additionally PT, APTT and TT, all of which were identified as coagulation factors, showed no statistical relationship with N stage in patients with stage T4a gastric cancer in the current study (**Supplementary Figure 2**).

Finally, multinomial logistic regression analysis identified tumor length, histopathologic grade, vascular invasion, and MA value as independent influencing factors for lymph node metastasis (**Table 5**). MA value was identified as a coagulation-related factor (β=0.059, 95% CI 0.015-0.104, P=0.009).

### 3.4 Utility of the MA value in predicting lymph node metastasis in gastric cancer

**Figure 4:**
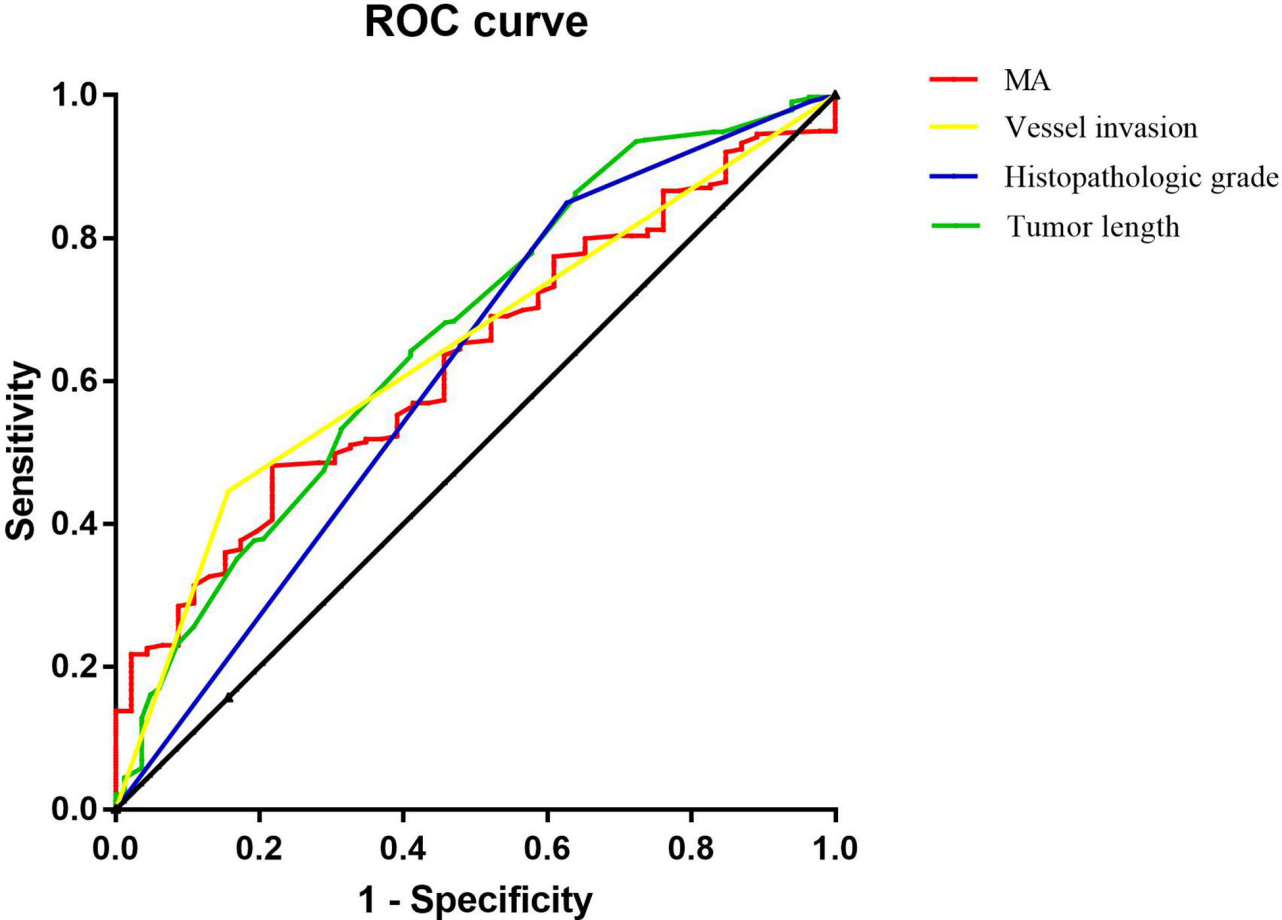
Figure of ROC analysis results of tumor length, histopathologic grade, vessel invasion and MA for predicting the lymph node metastasis of gastric cancer.

Binary logistic regression analysis showed that tumor length, histopathologic grade, vascular invasion, and MA value were independent influencing factors for lymph node metastasis in patients with T4a gastric cancer. ROC curve analysis was used to calculate the value of MA, a coagulation-related factor, in predicting lymph node metastasis in patients with gastric cancer (AUC=0.627, P=0.006, 95% CI: 0.548-0.707) (**Figure 4**, **Table 6**). The cut-off value for the MA value in predicting lymph node metastasis in patients with gastric cancer was 61.250 (sensitivity 0.477, specificity 0.783) (**Supplementary Table 1**).

**Table 6:** ROC analysis results of multiple factors for predicting the lymph node metastasis of gastric cancer.

Variables	Area	Sig	Asymptotic 95% Confidence Interval	
			Lower Bound	Upper Bound
Tumor length	0.684	0.000	0.602	0.767
Histopathologic grade	0.633	0.004	0.538	0.729
Vessel invasion	0.654	0.001	0.576	0.732
MA	0.627	0.006	0.548	0.707

### 3.5 Independent influencing factors for the MA value

**Figure 5:**
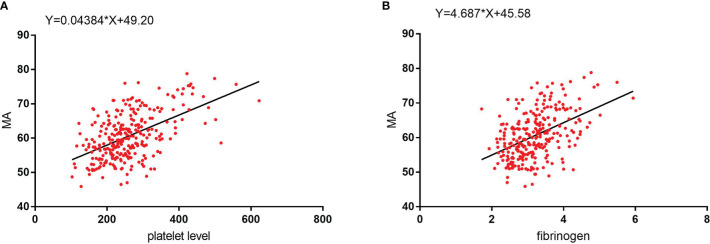
**(A)** MA values show a significant positive linear correlation with platelet level. **(B)** MA values show a significant positive linear correlation with fibrinogen level.

The influencing factors for the MA value were determined using univariate analysis. Stepwise linear regression was then used to confirm the independent influencing factors for MA, which included platelet (β=0.028, 95% CI: 0.019~0.036, P<0.001), fibrinogen (β=3.181, 95% CI 2.242~4.120, P<0.001), hemoglobin (β=-0.062, 95% CI: -0.089 ~ -0.035, P<0.001), and D-dimer levels (β=0.691, 95% CI: 0.116~1.265, P=0.019) (**Table 7**). We found that the MA value was positively, linearly, and statistically significantly correlated with both platelet counts and fibrinogen levels (**Figure 5**).

**Table 7:** Influencing factors of MA in patients with gastric cancer.

Variables	Univariate			Multivariate		
	EXP(β)	95%CI	p value	EXP(β)	95%CI	p value
Age	0.026	-0.051~0.103	0.510			
Sex	2.299	0.527~4.070	0.011			
ECOG	-1.224	-3.045~0.597	0.187			
ASA	-0.627	-1.964~0.710	0.357			
Tumor length	0.468	0.202~0.735	0.001			
Tumor short diameter	0.730	-0.034~1.495	0.061			
Tumor area	0.048	-0.008~0.103	0.091			
Primary tumor site	0.688	-0.226~1.603	0.140			
Histopathologic grade	0.177	-1.574~1.928	0.842			
WHO classification	0.798	-0.186~1.783	0.112			
Vessel invasion	1.733	0.135~3.331	0.034			
Neural invasion	-0.826	-2.437~0.784	0.313			
WBC	0.663	0.196~1.129	0.006			
RBC	-3.608	-4.740~-2.476	0.000			
HGB	-0.119	-0.147~-0.091	0.000	-0.062	-0.089~-0.035	0.000
Platelet	0.044	0.036~0.052	0.000	0.028	0.019~0.036	0.000
D-dimer	1.227	0.499~1.954	0.001	0.691	0.116~1.265	0.019
PT	0.817	-0.017~1.651	0.055			
APTT	-0.079	-0.304~0.145	0.486			
TT	-0.228	-0.843~0.388	0.467			
Fibrinogen	4.687	3.621~5.752	0.000	3.181	2.242~4.120	0.000

## 4 Discussion

In the field of oncological surgery, many studies conducted to date have found that components of the coagulation system may be involved in mediating the invasion and migration of malignant tumors (16–20). There are a number of studies that demonstrate the importance of haemostasis in the field of oncology (21). Moreover, some studies have reported correlations between coagulation-related factors and gastric cancer tumor stage (6, 7). There are no reports on the mechanisms of various coagulation-related factors in promoting lymph node metastasis in gastric cancer patients. To our knowledge, lymph node metastasis in gastric cancer patients has been related to various factors (e.g., tumor T stage and tumor histopathologic grade) (7, 22). In contrast to previous studies on the correlation between coagulation-related factors and tumor stage in patients with gastric cancer, our study is the first to exclude the effect of tumor T stage on lymph node metastasis in patients with gastric cancer. More specifically, we conducted the present analysis only among patients with stage T4a gastric cancer in order to analyze the correlation between coagulation-related factors and lymph node metastasis within gastric cancer. Our study clearly showed that lymph node metastasis in patients with T4a gastric cancer was statistically significantly correlated with multiple preoperative coagulation-related factors.

Among all 1128 initially included gastric cancer patients, 292 patients presented with T1 stage cancer, and the ratio of patients with N0 and N+ cancer was 5.49. The total number of patients with T2 stage cancer was n=151 and the total number of patients with T3 stage cancer was n=169 (**Table 3**). The proportion of patients at each N stage in patients with T1 stage cancer was not evenly distributed, and the number of patients with lymph node metastasis was extremely small. These factors limited the analysis of influencing factors for lymph node metastasis in gastric cancer within the present study.

Moreover, the total number of patients with T2 and T3 stage cancer was limited, and an analysis of lymph node metastasis in gastric cancer for T2 and T3 patients was therefore likely to be biased (i.e., with adverse effects on the accuracy of the resulting conclusions). Selecting patients with T1-T3 stage cancer for analysis would exert a large bias. Additionally, lymph node metastasis is affected by tumor T stage. Thus, in sum, we did not select all gastric cancer patients for analysis in the current study. Specifically, we selected a total 516 patients with T4a stage cancer (N0: 83, N1: 85, N2: 128, N3a: 210, N3b:10). The number of T4a patients enrolled in this study was large, as was the number of patients with lymph node metastasis. The proportion of gastric cancer patients under each N stage was moderate, and the analysis results were more robust. Therefore, we selected patients with T4a stage cancer to exclude the influence of T stage on lymph node metastasis in gastric cancer within the current investigation.

We found that the platelet level in the N+ group among patients with T4a gastric cancer was statistically significantly higher than that in the N0 group, and likewise detected a statistically significant relationship between platelet levels and lymph node metastasis in patients with gastric cancer. Moreover, we analyzed changes in platelet levels under different N stages, and found that platelet levels increased statistically significantly with N stage, as well as that platelet levels in patients at a low N stage were statistically significantly lower than those of patients with a higher N stage. Overall, our findings reflected a statistically significant positive correlation between tumor N stage and platelet levels in patients with T4a stage gastric cancer.

It was previously shown that platelets can enhance the malignant behavior of gastric cancer cells, including invasion and migration, through direct contact with gastric cancer cells (23). Other studies have found that antiplatelet drugs may play an important role in preventing gastric cancer progression, proliferation, and migration, reducing tumor cell metastasis, and reducing microvessel density (24). This further suggests that lymph node metastasis in patients with gastric cancer may be related to platelet counts. In contrast to previous studies, we evaluated only patients with T4a stage gastric cancer, thereby excluding the influence of tumor T stage. We found a statistically significant relationship between platelet levels and lymph node metastasis in patients with gastric cancer, as well as a positive correlation between tumor N stage and platelet levels.

The change trend in fibrinogen level was similar to that seen for platelet levels. In patients with T4a gastric cancer, fibrinogen levels in the N+ group were statistically significantly higher than those in the N0 group. Moreover, we found a statistically significant increase in fibrinogen levels with a higher tumor N stage. Overall, we found a statistically significant relationship between fibrinogen levels and lymph node metastasis in patients with T4a stage gastric cancer, as well as a positive correlation between the degree of lymph node metastasis and fibrinogen levels.

Fibrinogen may promote the metastasis of circulating tumor cells through several possible mechanisms. First, as a dimeric molecule with multiple integrin and non-integrin binding motifs, fibrinogen may serve as an important molecular bridge between tumor cells, platelets, and endothelial cells, thereby promoting stable adhesion (25). Second, fibrinogen-like protein 1 may promote the proliferation, invasion, and migration of gastric cancer cells by participating in the epithelial-mesenchymal transition (EMT) of cells (26). In addition, a lack of fibrinogen strongly reduces the spontaneous metastatic potential of aggressive tumor cell lines, including through blood and lymphatic pathways (27). Moreover, hyperfibrinogenemia may promote lymphatic metastasis in advanced gastric cancer and may be a useful predictor of lymphatic metastasis in gastric cancer (19, 20). Previous studies have found that fibrinogen is associated with lymph node metastasis and tumor stage in patients with gastric cancer (28). A large clinical trial also showed that preoperative serum fibrinogen levels were positively correlated with lymph node metastasis, tumor stage, and poor survival in patients with gastric cancer (6). The results of these studies all reflect the correlation between fibrinogen level and lymph node metastasis in patients with gastric cancer. However, previous studies did not consider the effect of tumor T stage when discussing the relationship between fibrinogen level and lymph node metastasis in gastric cancer. In contrast to previous studies, we evaluated only patients with T4a gastric cancer to exclude the effect of tumor T stage and found a statistically significant relationship between fibrinogen levels and lymph node metastasis in these patients, as well as a positive correlation between fibrinogen levels and the number of lymph node metastases.

Correlations between thromboelastography (TEG) parameters and routine test findings (including D-dimer, fibrinogen, and platelet levels) are consistent with a patient’s hypercoagulable state (29). TEG is widely used to assess the strength of platelet-fibrinogen interaction in forming clots, a function mainly represented by MA (30–33). In addition, MA values not only show statistically significant positive correlations with platelet levels, but also reflect the aggregation function of platelets (34, 35). Previous studies have demonstrated the presence of a strong linear positive correlation between MA values on TEG and both platelet and fibrinogen levels (36, 37). These previous results are consistent with our findings. In summary, the MA value is an indicator of the strength of blood clots formed by platelets and fibrinogen; MA mainly depends on the number of platelets and the aggregation function of platelets, and is also influenced by fibrinogen (30–37).

Our study showed that, in patients with T4a stage gastric cancer, the change trend for MA was similar to that of platelets and fibrinogen, and that the MA value of the N+ group was statistically significantly higher than that of the N0 group. MA statistically significantly increased with an increase in tumor N stage. In the binary logistic regression analysis comparing the N0 and N+ groups, we found that the MA value was an independent influencing factor for the presence or absence of lymph node metastasis in patients with T4a gastric cancer. Moreover, in the multinomial logistic regression analysis evaluating the MA value under each N stage, we continued to find that the MA value was an independent influencing factor for tumor N stage in these patients. The results of studies conducted to date have demonstrated a statistically significant positive correlation between the MA value derived from preoperative TEG in patients with T4a gastric cancer and the presence or absence of lymph node metastasis as well as the number of lymph node metastases in patients with gastric cancer. At the same time, the MA value was also an independent influencing factor for lymph node metastasis and tumor N stage in patients with T4a stage gastric cancer. The MA value is determined according to the combined effect of platelet levels, aggregation function, and fibrinogen levels. Research on the MA value has further confirmed the relationship between preoperative platelets, fibrinogen levels, and tumor lymph node metastasis in patients with gastric cancer.

All the above results indicated that the combined effect of platelet levels and aggregation function with fibrinogen in patients with gastric cancer is closely related to lymph node metastasis. ROC curve analysis determined that the cut-off value for MA in predicting the presence or absence of lymph node metastasis in patients with gastric cancer was 61.250 (sensitivity 0.477, specificity 0.783, P=0.006, 95% CI 0.548-0.707), and that the MA value is a reliable indicator for predicting the presence or absence of lymph node metastasis in patients with gastric cancer.

In their calm state, the surface of platelets is covered with a large number of membrane proteins (GPIIb/IIIa), all of which are in a resting state. When platelets are stimulated by signaling pathways, the GPIIb/IIIa on the platelet membrane is activated to connect with fibrinogen in order to achieve platelet activation (8, 38). Therefore, platelet activation is inseparable from the interaction between platelets and fibrinogen. It has previously been reported that the MA value is related to the level and aggregation function of platelets as well as to fibrinogen levels (30–37). Our study also showed that platelet and fibrinogen levels were independent influencing factors for the MA value. Therefore, the MA value ​​can reflect platelet activation and fibrinogen level.

Moreover, basic research has shown that platelet activation and the presence of fibrinogen helps tumor cells evade immune surveillance mechanisms and protecting tumor cells from natural killer cells (NK cells), including through physical means and signals that cause NK cells to quiesce (25, 39, 40). Therefore, gastric cancer cells can survive in blood and body fluids as circulating tumor cells (CTCs), causing biological behaviors such as hematogenous metastasis and lymphatic metastasis in gastric cancer. Meanwhile, activated platelets express CD40 ligand (CD40L) and CD62P in the cytoplasmic membrane, and the interaction with the vascular endothelium may induce tumor growth factor production and tumor metastasis (15). The abovementioned studies all reflect the promoting effect of platelet activation on the malignant behavior of tumor cells, and may also explain why the MA value is an independent factor for lymph node metastasis and tumor N stage in patients with gastric cancer.

In addition, D-dimer and hemoglobin levels were also independent influencing factors for the MA value. Patients with advanced gastric cancer are prone to chronic blood loss, reduced oral iron intake, and malabsorption at the tumor site (41). In our study, we found that as the tumor length increased, the patient’s blood loss continued to increase and hemoglobin continued to decrease (**Table 8**). Moreover, previous findings indicate that, as the body continues to lose blood, serotonin (which is transported by platelets) releases with platelet activation; this induces vasoconstriction at the bleeding site and enhances platelet activation in order to minimize blood loss (42). Enhanced platelet activation further results in an increase in clot strength, and hemoglobin becomes an independent and inversely related predictive factor for MA. Moreover, D-dimer is a stable end product of the degradation of cross-linked fibrin as a result of enhanced fibrin formation and fibrinolysis (43). When blood loss increases, the activation of the coagulation system is enhanced. At the same time, D-dimer levels increase along with hyperactivity of the fibrinolysis system. Therefore, D-dimer levels were found to be an independent influencing factor for MA values and were positively correlated with MA.

**Table 8:** The effect of tumor length on hemoglobin.

Variables	β	Sig	Asymptotic 95% Confidence Interval	
			Lower Bound	Upper Bound
Tumor length	-1.410	0.000	-2.199	-0.621

Our study found no statistically significant association between PT, APTT, or TT and lymph node metastasis in patients with T4a gastric cancer. PT mainly reflects the common pathway of the coagulation cascade and the extrinsic coagulation pathway, while APTT mainly reflects the common pathway of the coagulation cascade and the intrinsic coagulation pathway (44–46). TT mainly reflects the conversion of fibrinogen to fibrin (46). Together, these three coagulation factors reflect the function of the coagulation system and the coagulation process. Platelet activation is a necessary condition for the enhancement of coagulation function, but platelet activation cannot directly reflect the enhancement of coagulation function. Therefore, the MA value, which can reflect platelet activation and fibrinogen levels, is related to lymph node metastasis in gastric cancer. However, the enhancement of coagulation function shows no correlation with lymph node metastasis in gastric cancer.

Some previous studies have shown that the combined diagnosis of gastroscopy, MSCT, immunohistochemical marker Her-2, and tumor markers CEA, CA199, CA724, and CA242 can more accurately determine the clinical staging and lesion invasion depth of patients with gastric cancer and can significantly improve the sensitivity of diagnosis (47). A deep learning-based radiomic nomogram had good predictive value for lymph node metastasis in locally advanced gastric cancer (48). This suggests that there are a variety of ways to predict lymph node metastasis in gastric cancer. Our study provides additional reference for lymph node metastasis and is a useful addition to other methods of preoperative assessment of lymph node metastasis in patients with gastric cancer, such as imaging. This does not negate the important value of imaging, such as CT, and many other methods for the assessment of lymph node metastasis.

Coagulation factors are an important part of the clotting system and they play a role in the blood clotting process. However, as coagulation factors have not been routinely tested in our previous studies, they have not been explored further in this paper, which is a shortcoming of this study and a direction that deserves further investigation.

## 5 Conclusion

In patients with T4a gastric cancer, univariate analysis determined that platelet counts, fibrinogen levels, and MA values were statistically significantly positively correlated with lymph node metastasis and tumor N stage. Multivariate analysis found that the independent influencing factors for lymph node metastasis and tumor N stage in patients with T4a gastric cancer included tumor length, histopathologic grade, vessel invasion, and MA value, among which MA was a coagulation-related factor. Moreover, we found that the MA value effectively predicts the presence of lymph node metastasis in patients with gastric cancer. In addition, platelet, fibrinogen, D-dimer, and hemoglobin levels were independent influencing factors for MA.

## Data availability statement

The raw data supporting the conclusions of this article will be made available by the authors, without undue reservation.

## Ethics statement

This study was approved by the Ethics Committee of Shandong Provincial Hospital (SWYX : NO. 2022-466).

## Author contributions

WQ: writing - original draft, writing - review and editing, and formal analysis. SS: writing - original draft, and formal analysis. JS: writing - original draft, and data curation. YC: software. GL: resources. JW: data curation. XZ: data curation. LP: data curation. LL: resources. FT: methodology, conceptualization, supervision, and funding acquisition. CJ: methodology, conceptualization, supervision, and funding acquisition. All authors contributed to the article and approved the submitted version.
