# Supplementary material for: Association between multiple coagulation-related factors and lymph node metastasis in patients with gastric cancer: A retrospective cohort study
Source: Front Oncol. 2023 Feb 23;13:1099857. doi: 10.3389/fonc.2023.1099857 (PMC9996287; doi:10.3389/fonc.2023.1099857)
Supplement: Supplementary file 1 [file Image_1.pdf]

Supplementary Material

Supplementary Figures

Supplementary Figure 1 Comparison of differences in multiple coagulation-related factors according to whether lymph node metastases or not. a. Lymph node metastasis in patients with gastric cancer is not related to D-dimer. b. Lymph node metastasis in patients with gastric cancer is not related to PT. c. Lymph node metastasis in patients with gastric cancer is not related to APTT. d. Lymph node metastasis in patients with gastric cancer is not related to TT.

Supplementary Figure 2 Comparison of differences in multiple coagulation-related factors according to tumor N stage of patients with gastric cancer. a. Tumor N stage of patients with gastric cancer is not related to PT. b. Tumor N stage of patients with gastric cancer is not related to APTT. c. Tumor N stage of patients with gastric cancer is not related to TT.

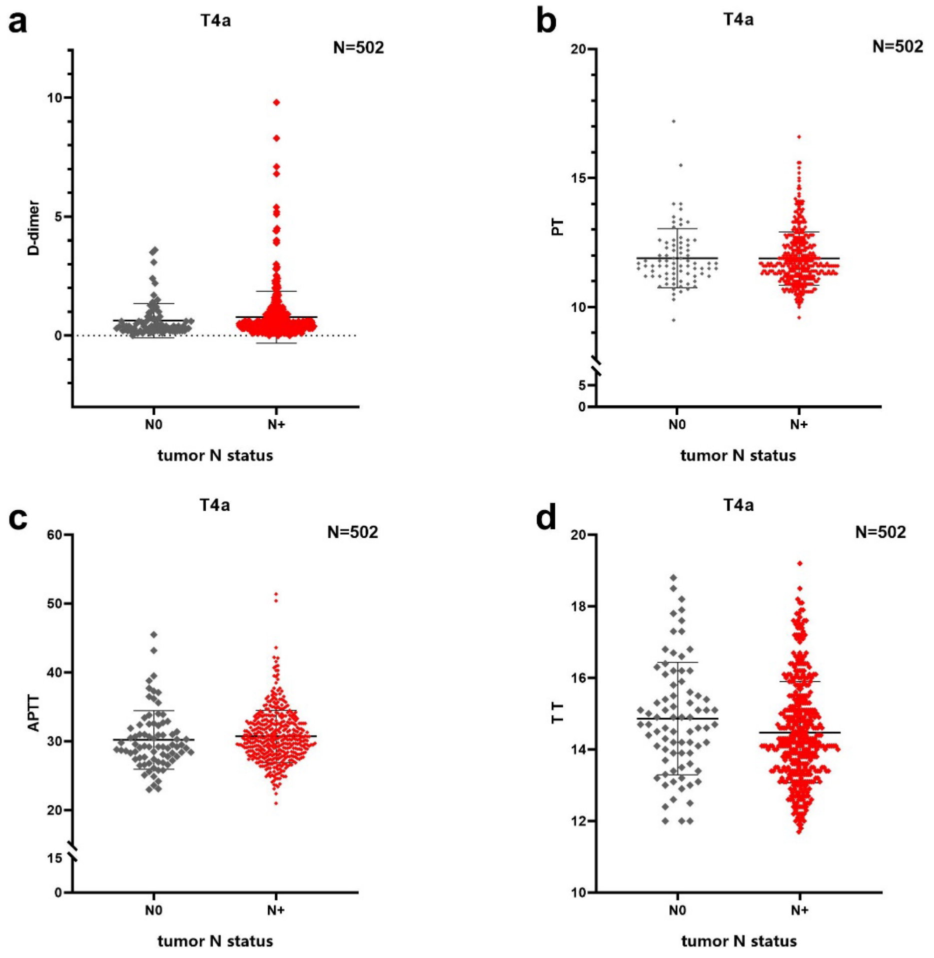

Supplementary Figure 1 Comparison of differences in multiple coagulation-related factors according to whether lymph node metastases or not. **a.** Lymph node metastasis in patients with gastric cancer is not related to D-dimer. **b.** Lymph node metastasis in patients with gastric cancer is not related to PT. **c.** Lymph node metastasis in patients with gastric cancer is not related to APTT. **d.** Lymph node metastasis in patients with gastric cancer is not related to TT.

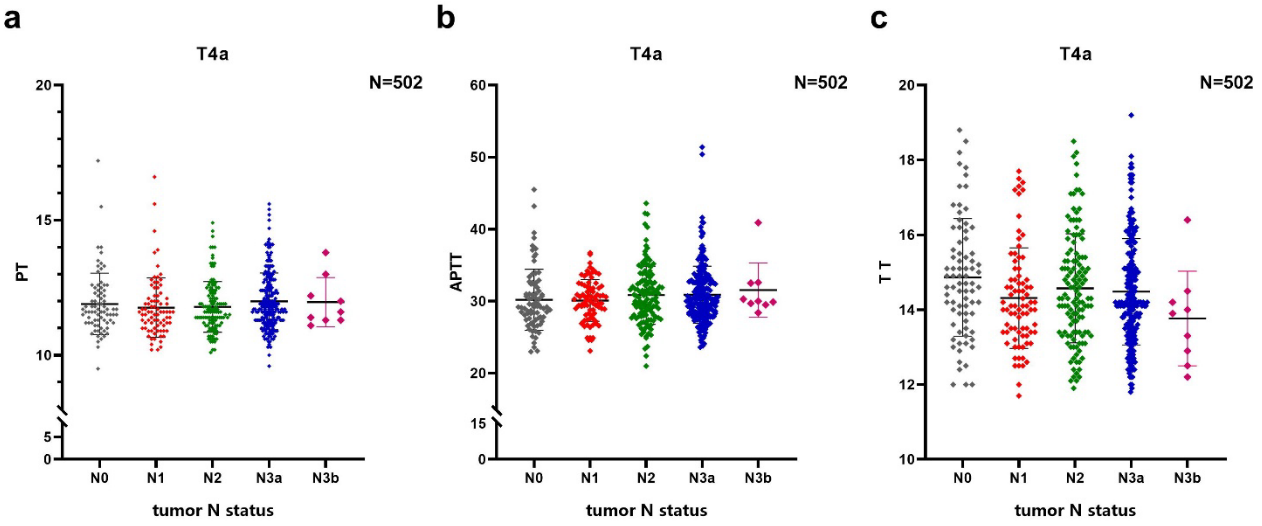

Supplementary Figure 2 Comparison of differences in multiple coagulation-related factors according to tumor N stage of patients with gastric cancer. **a.** Tumor N stage of patients with gastric cancer is not related to PT. **b.** Tumor N stage of patients with gastric cancer is not related to APTT. **c.** Tumor N stage of patients with gastric cancer is not related to TT.
